# Up-regulation of Orai1 expression and store operated Ca^2+^ entry following activation of membrane androgen receptors in MCF-7 breast tumor cells

**DOI:** 10.1186/s12885-015-2014-2

**Published:** 2015-12-21

**Authors:** Guilai Liu, Sabina Honisch, Guoxing Liu, Sebastian Schmidt, Saad Alkahtani, Abdullah A. AlKahtane, Christos Stournaras, Florian Lang

**Affiliations:** 1 Department of Physiology, University of Tuebingen, Tuebingen, Germany; 2 Department of Biochemistry, University of Crete Medical School, Heraklion, Crete Greece; 3 Department of Zoology, Science College, King Saud University, Riyadh, Saudi Arabia; 4 Physiologisches Institut, der Universität Tübingen, Gmelinstr. 5, D-72076 Tübingen, Germany

**Keywords:** Ca^2+^ release activated Ca^2+^ channel, SOCE, 2-APB, Cytochalasin B, Rac-1

## Abstract

**Background:**

Membrane androgen receptors (mAR) are functionally expressed in a variety of tumor-cells including the breast tumor-cell line MCF-7. They are specifically activated by testosterone albumin conjugates (TAC). The mAR sensitive signaling includes activation of Ras-related C3 botulinum toxin substrate 1 (Rac1) and reorganization of the actin filament network. Signaling of tumor-cells may further involve up-regulation of pore forming Ca^2+^ channel protein Orai1, which accomplishes store operated Ca^2+^ entry (SOCE). This study explored the regulation of Orai1 abundance and SOCE by mAR.

**Methods:**

Actin filaments were visualized utilizing confocal microscopy, Rac1 activity using GST-GBD assay, Orai1 transcript levels by RT-PCR and total protein abundance by western blotting, Orai1 abundance at the cell surface by confocal microscopy and FACS-analysis, cytosolic Ca^2+^ activity ([Ca^2+^]_i_) utilizing Fura-2-fluorescence, and SOCE from increase of [Ca^2+^]_i_ following readdition of Ca^2+^ after store depletion with thapsigargin (1 μM).

**Results:**

TAC treatment of MCF-7 cells was followed by Rac1 activation, actin polymerization, transient increase of Orai1transcript levels and protein abundance, and transient increase of SOCE. The transient increase of Orai1 protein abundance was abrogated by Rac1 inhibitor NSC23766 (50 μM) and by prevention of actin reorganization with cytochalasin B (1 μM).

**Conclusions:**

mAR sensitive Rac1 activation and actin reorganization contribute to the regulation of Orai1 protein abundance and SOCE.

## Background

Membrane androgen receptors (mARs) are functionally expressed in various tumor cells including prostate [1–6], breast [6–9] and colon cancer cells [10, 11] as well as gliomas [12]. The mARs are specifically activated by membrane impermeable testosterone albumin conjugates (TAC) [5, 13, 14]. Signaling mediating the cellular effects of mAR’s include the early FAK/PI3K/SGK1/Rac1/Cdc42 and Rho/ROCK/LimK cascades and late GSK/beta-catenin pathway leading to profound actin reorganization [5, 7, 9, 14–18]. Activation of mARs eventually leads to modification of tumor cell proliferation, migration and apoptosis [5, 6, 13, 14, 19].

Cell proliferation, migration and cell death are regulated by alterations of cytosolic Ca^2+^ activity [20–23]. A powerful regulator of cytosolic Ca^2+^ concentration is the pore forming Ca^2+^ channel subunit Orai1 accomplishing store operated Ca^2+^ entry (SOCE) [24–30]. In a recent study, activation of mAR by dehydrotestosterone (DHT) in prostate cancer cells was reported to induce rapid Ca^2+^ influx via Orai that was important for rapid androgen effects [31].

The present study explored, whether mAR activation is followed by alterations of Orai1 protein abundance and function in MCF-7 breast tumor cells and addressed the role of actin reorganization and actin signaling to this effect. To this end, MCF-7 cells were exposed to TAC and Orai1 protein abundance at the cell surface was determined by confocal microscopy and flow cytometry as well as intracellular Ca^2+^ release and SOCE were quantified utilizing Fura-2 fluorescence.

## Results and discussion

**Fig. 1:**
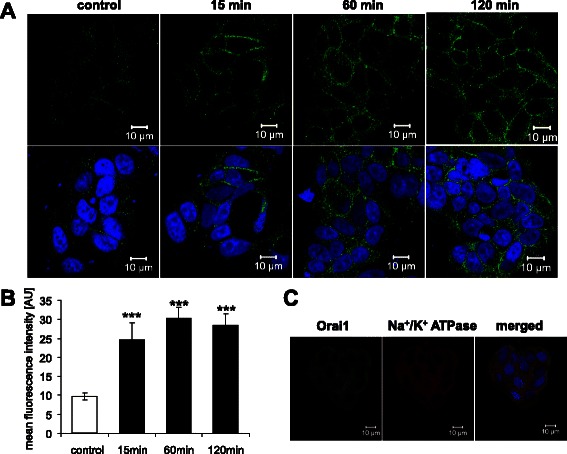
Effect of mAR activation on Orai1 protein abundance at the surface of MCF-7 cells. **a** Original confocal microscopy of non-permeabilized MCF7 cells treated for 15–120 min with TAC-BSA (100 nM) and stained with anti-Orai1 antibody (green) and DRAQ-5 (blue) for nuclei. **b** Arithmetic means ± SEM (*n* = 6) of Orai1 protein abundance in non-permeabilized MCF-7 cells without (white bar) and with (black bars) a 15 min to 120 min treatment with testosterone-albumin-conjugates (TAC, 100 nM). ***(*p* < 0.001) indicates statistically significant difference from absence of TAC. **c** Original confocal microscopy demonstrating colocalization of Orai1 (green) and Na^+^/K^+^ ATPase (red) in MCF7 cells. DRAQ-5 (blue) indicates nuclei

**Fig. 2:**
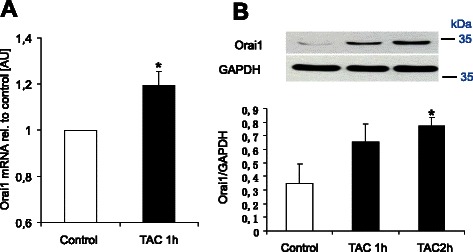
Effect of mAR activation on Orai1 transcript levels and total protein abundance. **a** Arithmetic means ± SEM (*n* = 6) of Orai 1 transcript levels as determined by RT-PCR in MCF-7 cells without (white bar) and with (black bars) a 60 min treatment with testosterone-albumin-conjugates (TAC, 100 nM). *(*p* < 0.05) indicates statistically significant difference from absence of TAC. **b** Original Western blot and arithmetic means ± SEM (*n* = 3) of protein abundance in MCF-7 cells without (white bar) and with (black bars) a 60 min and 120 min treatment with testosterone-albumin-conjugates (TAC, 100 nM). *(*p* < 0.05) indicates statistically significant difference from absence of TAC

The present study explored the effect of membrane androgen receptor (mAR) activation on Ca^2+^ signaling in MCF-7 breast cancer cells. To this end, MCF- cells were treated with testosterone-albumin conjugates (TAC, 100 nM), which selectively activate mAR without activating intracellular androgen receptors (iAR). In a first step, confocal microscopy was employed to visualize the effect of TAC on the Orai1 protein abundance at the MCF-7 cell membrane surface. As illustrated in Fig. 1, mAR activation was followed by a rapid increase of Orai1 protein abundance at the surface of MCF-7 cells. Quantitative analysis revealed that mAR activation significantly increased Orai1 abundance within 15 min, an effect that was persistent for at least 120 min (Fig. 1b). Orai1 was colocalized with Na^+^/K^+^ ATPase (Fig. 1c). In contrast to Orai1 abundance, the Na^+^/K^+^ ATPase protein abundance was similar without mAR activation (24.5 ± 1.1 a.u., *n* = 5) and 1 h (24.7 ± 2.2 a.u., *n* = 5) or 2 h (25.0 ± 1.9 a.u., *n* = 5) following mAR activation. The increased Orai1 abundance in the cell membrane was paralleled by an increase of Orai1 transcript levels (Fig. 2a) and protein abundance (Fig. 2b).

**Fig. 3:**
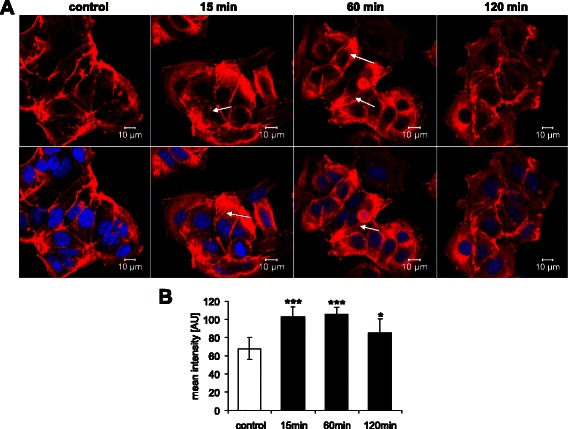
Modulation of dynamic actin polymerization by mAR activation of MCF-7 cells. **a** Original confocal images of rhodamine-phalloidin binding to F-actin (red) and DRAQ-5 for nuclei (blue) in MCF-7 cells without (control) and with a prior 15–120 min treatment with testosterone-albumin-conjugates (TAC, 100 nM). Arrows point to formation of actin stress fibers. **b** Arithmetic means ± SEM (*n* = 6) of actin fluorescence in MCF-7 cells without (white bar) and with (black bars) a 15 min to 120 min treatment with testosterone-albumin-conjugates (TAC, 100 nM). *(*p* < 0.05) and ***(*p* < 0.001) indicate statistically significant difference from absence of TAC

**Fig. 4:**
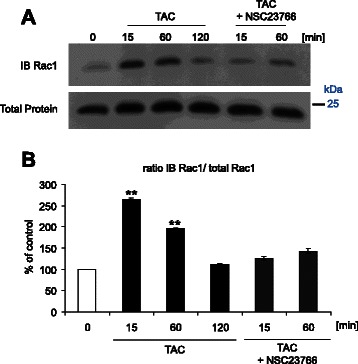
Effect of mAR activation on abundance of activated Rac1 protein in MCF-7 cells. **a** Affinity precipitation with GST (glutatione S-transferase) -PBD (p21 binding domain) revealing by immunoblotting (IB) the protein abundance of activated (upper lane) and total (lower lane) Rac1 prior to (control) and 15–120 min following treatment with testosterone-albumin-conjugates (TAC, 100 nM) and TAC + Rac1 inhibitor NSC23766 (50 μM). **b** Arithmetic means ± SEM (*n* = 4) of the relative fold increases of activated over total Rac1 protein abundance prior to (taken as 1) and 15–120 min following treatment with testosterone-albumin-conjugates (TAC, 100 nM) and TAC + Rac1 inhibitor NSC23766 (50 μM). **(*p* < 0.01) indicates statistically significant difference from absence of TAC

As shown in Fig. 3, the effect of mAR activation on Orai1 abundance was paralleled by a profound reorganization of the actin cytoskeleton of MCF-7 cells. According to Fig. 4, the treatment of MCF-7 cells with TAC was followed by rapid and transient activation of the Rac1 protein, an effect abrogated by the specific Rac1 inhibitor NSC23766 (50 μM).

**Fig. 5:**
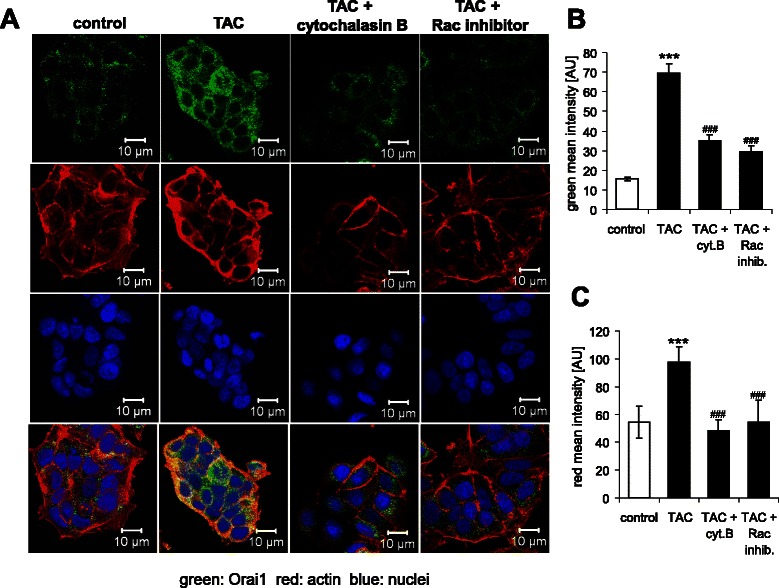
Effect of mAR activation on actin cytoskeleton and membrane Orai1 abundance of MCF-7 cells. **a** Original confocal microscopy of actin filaments (red) and Orai1 (green) in non-permeabilized MCF-7 cells without (control) and with a prior 60 min treatment with testosterone-albumin-conjugates (TAC, 100 nM) alone (TAC) or together with cytochalasin B (1 μM) (TAC + cytochalasin B), or with Rac inhibitor NSC23766 (50 μM) (TAC+ Rac inhibitor). **b**, **c** Arithmetic means ± SEM of (**b**) Orai1 abundance (*n* = 6) and of (**c**) actin fluorescence (*n* = 6) in MCF-7 cells without (white bar) and with (black bars) a 60 min treatment with testosterone-albumin-conjugates (TAC, 100 nM). ***(*p* < 0.001) indicates statistically significant difference from absence of TAC, ###(*p* < 0.001) indicates statistically significant difference from presence of TAC without presence of inhibitors

As illustrated in Fig. 5, the effect of mAR activation on Orai1 abundance of MCF-7 cells was prevented by presence of each, cytochalasin B (1 μM) and Rac inhibitor NSC23766 (50 μM) (TAC+ Rac inhibitor), suggesting that actin reorganization may represent an important Orai1-regulator.

**Fig. 6:**
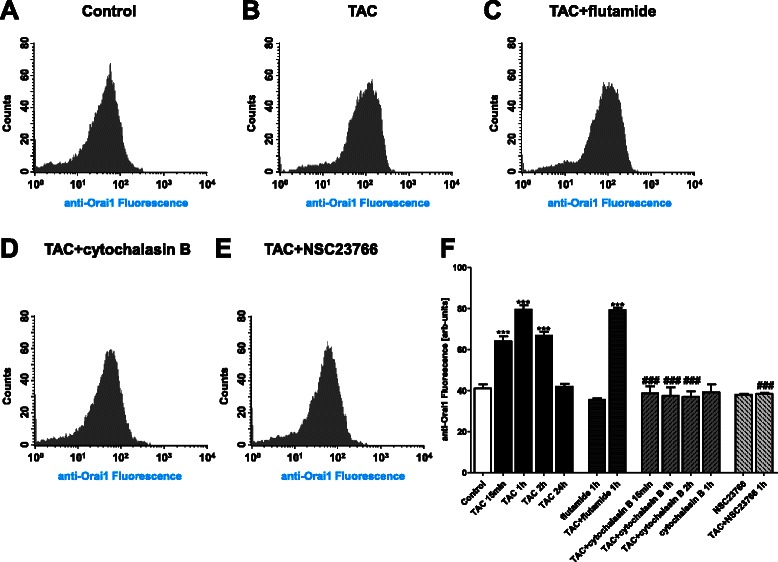
Total Orai1 abundance in MCF-7 cells following mAR activation in absence and presence of cytochalasin B and Rac1 inhibitor NSC23766. **a**-**e** Original histogram of anti-Orai1 fluorescence in permeabilized MCF-7 cells without (**a**) and with (**b**-**e**) a 60 min treatment with testosterone-albumin-conjugates (TAC, 100 nM) in the absence (**b**) and presence of flutamide (**c**), cytochalasin B (**d**) and Rac inhibitor (**e**). **f** Arithmetic means ± SEM (*n* = 6) of the Orai 1 protein abundance in permeabilized MCF-7 cells without (white bar) and with a 15 min to 24 h treatment with testosterone-albumin-conjugates (TAC, 100 nM) in the absence (black bars) and presence of flutamide (1 μM) (dark grey bars) cytochalasin B (1 μM) (middle grey bars), or Rac inhibitor NSC23766 (50 μM) (light grey bars). ***(*p* < 0.001) indicates statistically significant difference from absence of TAC, ###(*p* < 0.001) indicates statistically significant difference from presence of TAC without presence of inhibitors

**Fig. 7:**
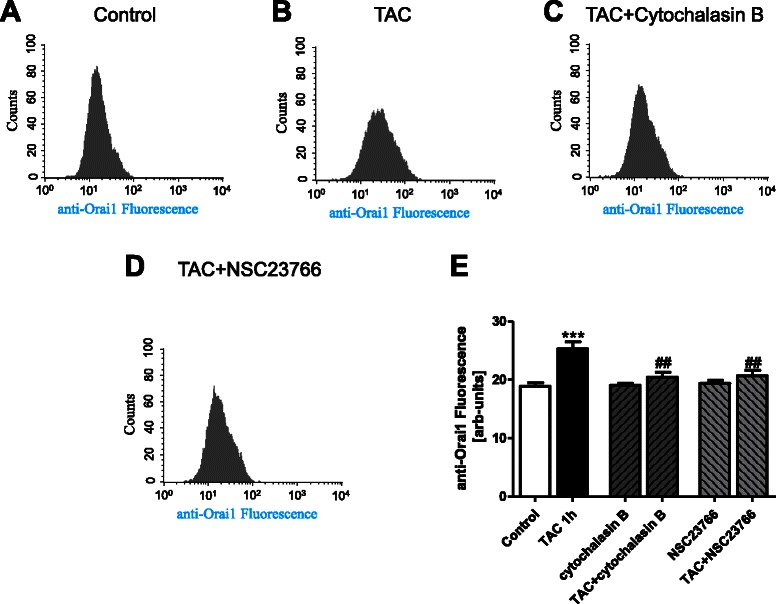
Cell membrane Orai1 abundance in MCF-7 cells following mAR activation in absence and presence of cytochalasin B and Rac1 inhibitor NSC23766. **a**-**d** Original histogram of anti-Orai1 fluorescence in non-permeabilized MCF-7 cells without (**a**) and with (**b**-**d**) a 60 min treatment with testosterone-albumin-conjugates (TAC, 100 nM) in the absence (**b**) and presence of cytochalasin B (**c**) and Rac inhibitor (**d**). **e** Arithmetic means ± SEM (*n* = 6) of the Orai 1 protein abundance in non-permeabilized MCF-7 cells without (white bar) and with a 60 min treatment with testosterone-albumin-conjugates (TAC, 100 nM) in the absence (black bar) and presence of cytochalasin B (1 μM) (middle grey bars), or Rac inhibitor NSC23766 (50 μM) (light grey bars). ***(*p* < 0.001) indicates statistically significant difference from absence of TAC, ##(*p* < 0.01) indicates statistically significant difference from presence of TAC without presence of inhibitors

Flow cytometry was employed to further quantify the alterations of Orai1 protein abundance at the MCF-7 cell surface. The TAC treatment of MCF-7 cells was followed by a transient increase of the Orai1 protein abundance in MCF-7 cells (Fig. 6). The effect of TAC on Orai1 protein abundance at the MCF-7 cell surface was not significantly modified by the intracellular androgen receptor blocker flutamide (1 μM), but was virtually abrogated in the presence of cytochalasin B (1 μM) or the presence of Rac inhibitor NSC23766 (50 μM). Similar results were obtained in non-permeabilized cells (Fig. 7).

**Fig. 8:**
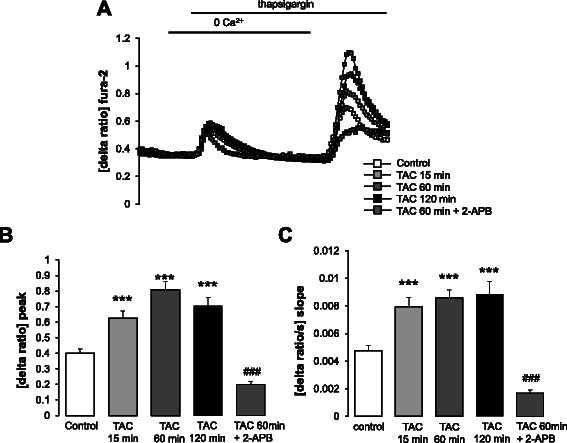
Effect of mAR activation on intracellular Ca^2+^ release and store operated Ca^2+^ entry (SOCE) in MCF-7 cells. **a** Representative tracings of fura-2 fluorescence-ratio in fluorescence spectrometry before, during and after Ca^2+^ depletion with subsequent addition of thapsigargin (1 μM) in MCF-7 cells without (control, open squares) and with (grey and black squares) treatment with testosterone-albumin-conjugates (TAC, 100 nM) for 15–120 min in the absence and presence of the Orai-1 inhibitor 2-APB (50 μM). **b**, **c** Arithmetic means (± SEM, *n* = 3–5, each experiment 10–30 cells) of slope (**b**) and peak (**c**) increase of fura-2-fluorescence-ratio following re-addition of extracellular Ca^2+^ in MCF-7 cells without (control, white bars) and with (grey and black bars) treatment with TAC (100 nM) for 15–120 min in the absence and presence of the Orai-1 inhibitor 2-APB (50 μM). ***(*p* < 0.001) indicates statistically significant difference from absence of TAC, ###(*p* < 0.001) indicates statistically significant difference from 60 min presence of TAC without presence of 2-APB (ANOVA)

Fura-2 fluorescence was employed to quantify alterations of cytosolic Ca^2+^ activity ([Ca^2+^]_i_). The store operated Ca^2+^ entry (SOCE) was apparent from increase of [Ca^2+^]_i_ following readdition of extracellular Ca^2+^ after store depletion with the sarcoendoplasmatic reticulum Ca^2+^ ATPase (SERCA) inhibitor thapsigargin (1 μM). As illustrated in Fig. 8, TAC treatment had little effect on thapsigargin-induced intracellular Ca^2+^ release but was followed by a marked transient increase of both, slope and peak, of SOCE in MCF-7 cells. SOCE was virtually disrupted by the Orai1 inhibitor 2-APB (50 μM).

The present study reveals that activation of membrane androgen receptors (mARs) by testosterone albumin conjugates (TAC) triggers a strong transient increase of Orai1 protein abundance in the MCF-7 breast cancer cell surface. This effect was not dependent on the intracellular androgen receptor, as shown by control experiments in the presence of the anti-androgen drug flutamide. It was paralleled by and presumably accounted for a profound increase of store operated Ca^2+^ entry. The effects required activation of Rac1 GTPase and reorganization of the actin cytoskeleton. Accordingly, the up-regulation of Orai1 was virtually abrogated by Rac1 inhibitor NSC23766 and by disruption of the actin filament network with cytochalasin B.

Orai1 contributes to the regulation of cell proliferation [32–37]. Stimulation of SOCE triggers Ca^2+^ oscillations [38, 39], which influence a wide variety of cellular functions [40–44]. Notably, the Ca^2+^ oscillations trigger depolymerization of actin filaments [40, 45]. As depolymerization of the actin filaments disrupts the effect of mARs on Orai1 protein abundance, it is tempting to speculate that it is the Ca^2+^-induced depolymerization of the actin filaments, which leads to the transient nature of the TAC effect. At least in some cells, Ca^2+^ oscillations and actin depolymerization are required for the stimulation of cell proliferation [40].

Actin reorganization following mARs activation, regulated by various actin signaling pathways [46] modifies several cellular functions including stimulation of apoptosis [10, 11, 14, 47] and migration [7, 11, 16]. The mAR-induced apoptotic response in breast cancer cells is disrupted by the actin cytoskeleton inhibitor cytochalasin B that blocks the observed actin reorganization [9]. Similar to the effect of mAR activation on Orai1 abundance, the effect of mAR on apoptosis requires actin polymerization. Thus, actin reorganization is a pivotal response of cells to activation of mARs, as well as to effects on apoptosis, cell death and aging [13, 48–52]. The mechanism by which Orai 1 abundance may be regulated by the Rac1 governed actin reorganization remains to be elucidated. Regulation of ORAI1 gene transcription may well involve early actin redistribution, as this was previously reported for the transcription of various genes encoding specific regulatory effectors [53–55]. Moreover, the cytoskeleton may impact on trafficking of expressed Orai1 protein to the cell membrane [56–59]. However, additional experimental efforts are required to fully understand the complex signaling of mAR induced cellular functions and death [46].

## Conclusions

In conclusion, transient up-regulation of Orai1 protein abundance and transient increase of store operated Ca^2+^ entry contribute to the signaling of the membrane androgen receptors. Actin reorganization, regulated by mAR-induced early Rac1 GTPase activation is involved in the regulation of Orai1 protein abundance and SOCE. Thus mAR participates in the regulation of Ca^2+^ signaling.

## Methods

### Cell culture

MCF-7 mammary adenocarcinoma cells, provided from ATCC were cultured in DMEM high glucose medium (Gibco) containing 10 % FBS and 1 % penicillin/streptomycin in a humidified atmosphere of 5 % CO_2_. Based on previous titration experiments [2, 7, 10] for mAR stimulation, we have used throughout this study the non-permeable androgen derivative testosterone-BSA (TAC, Sigma-Aldrich) in a concentration of 100 nM. In some experiments, the anti-androgen drug flutamide (1μM, Sigma), the Rac1 inhibitor NSC23766 (50 μM) or the actin cytoskeleton-disrupting agent cytochalasin B (1μM, Sigma-Aldrich) were used as indicated.

### Confocal laser scanning microscopy

For actin, Orai1 and Na^+^K^+^ATPase staining, 7 × 10^4^ MCF-7 cells were cultured for 24 h on glass cover slips and treated or not with TAC-BSA (100 nM, Sigma), cytochalasin B (1μM, AppliChem) and Rac1 inhibitor NSC23766 (50 μM) for different time periods as indicated in the figure legends. After washing twice with PBS, cells were fixed with 4 % PFA for 15 min and then blocked with 3 % BSA in PBS for 1 h at room temperature. Then, the cells were exposed to anti-Orai1 primary antibody (1:200, Abcam #ab 59330) or/and anti- Na^+^K^+^ATPase (Sigma, USA) at 4 °C overnight. The cells were rinsed three times with PBS and incubated with secondary antibody for Orai1 CF™ 488A-labeled anti–rabbit (1:250, Sigma, USA) and for Na^+^K^+^ATPase CF™ 555-labeled anti-mouse antibody (1:250, Sigma, USA) for 1 h at room temperature. Additional cells were incubated with rhodamine-phalloidin (1:200, Life Technologies, USA) for F-actin and with DRAQ-5 dye (1:3000, Biostatus, Leicestershire, UK) for nuclei staining for 30 min in the dark. All slides were mounted with ProLong Gold antifade reagent (Life Technologies, USA). Images were subsequently taken on a Zeiss LSM 5 EXCITER confocal laser scanning microscope (Carl Zeiss, Germany) with a water immersion Plan-Neofluar 63/1.3 NA DIC [60, 61]. The mean fluorescence from six related cells of each picture was quantified by ZEN software (Carl Zeiss, Germany).

### Quantitative RT-PCR

To determine Orai1 gene expression, MCF7 cells were washed twice with PBS, and lysed with 1ml TriFast Reagent (Peqlab, Erlangen, Germany). The RNA was isolated according to the manufacturer’s protocol. 2.5 μg of the RNA were transcribed to cDNA using the GoScript™ Reverse Transcription System (Promega Corporation, Madison, USA) and oligo-dT primers. Quantitative real-time PCR was performed on the CFX96 cycler (Bio-Rad) in a total volume of 20 μl using 2 μl of cDNA, and 2x GoTaq® qPCR Master Mix (Promega Corporation, Madison, USA). Cycling conditions were initial denaturation at 95 °C for 5 min, followed by 40 cycles of 95 °C for 15 s, 59 °C for 30 s and 72 °C for 30 s.

The following primers were used (5′- > 3′orientation):

ORAI1 forward primer: AGCCTCAACGAGCATCCCAT

ORAI1 forward reverse primer: CTGATCATGAGCGCAAACAGG

GAPDH forward primer: TGAGTACGTCGTGGAGTCCACTG

GAPDH reverse primer: CACCACCAACTGCTTAGCACC

Relative quantification of the gene expression was achieved using the ∆∆Ct method and GAPDH as housekeeping gene.

### Western blotting

Cells were incubated with with TAC-BSA (100 nM, Sigma) for the indicated time periods, washed twice with ice-cold PBS and suspended in ice-cold lysis buffer (50 mM Tris/HCl, 1 % TritonX-100 pH 7.4, 1 % sodium deoxycholate, 0.1 % SDS, 0.15 % NaCl, 1 mM EDTA, 1 mM sodium orthovanadate) containing a protease inhibitor cocktail (Sigma). The protein concentration was determined using the Bradford assay (BioRad). 40 μg of total proteins were boiled with Roti-Load sample buffer (Carl Roth, Germany) for 5 min at 95 °C and separated in 10 % SDS-PAGE. Proteins were transferred to a PVDF-membrane (Thermo Fisher Scientific, USA) and blocked for 1 h at room temperature with 5 % BSA (Carl Roth, Germany) in TBST. For immunostaining membranes were incubated overnight at 4 °C with anti-Orai1 (1:1000, Cell Signaling) and GAPDH (1:3000, Cell Signaling, USA) antibodies. To detect the specific proteins membranes were incubated for 1h at RT with a 1:2000 dilution of anti-rabbit IgG conjugated to horseradish peroxidase (Cell Signaling, USA). After washing, bands were visualized using the ECL western blotting detection reagent (GE Healthcare, USA) and quantified by Quantity One Software (ChemiDoc XRS, Bio-Rad, USA).

### Rac1 activity

Rac1 activity was determined utilizing affinity precipitation with GST-PBD as described previously [62]. In brief, cells, treated or not with TAC (100 nM) in the presence or absence of the specific Rac1 inhibitor NSC23766 (50 μM) were lysed in Mg^2+^ lysis buffer (Upstate Biotechnology, Inc.) and incubated with 200 μl of binding buffer composed of (all in mM) 25 Tris–HCl (pH 7.5), 1 DTT, 30 MgCl_2_, 40 NaCl, and with added 0.5 % Nonidet P-40, and 5 μl glutathione-Sepharose 4B beads at 4 °C. The bead pellet was then washed 3 times with a buffer composed of (in mM) 25 Tris–HCl (pH 7.5), 1 DTT, 30 MgCl_2_, 40 NaCl, with or without added 1 % Nonidet P-40. The bead pellet was finally suspended in 20 μl of Laemmli sample buffer. Proteins were separated by 11 % SDS-PAGE, transferred onto nitro-cellulose membrane, and immunoblotted with anti-Rac1 antibody (1:1000, Cell Signaling, USA).

### FACS analysis of Orai1 surface and total protein abundance

Orai1 surface expression was analyzed by flow cytometry. To this end, the cells were detached, washed three times with phosphate-buffered saline (PBS) and fixed with 4 % paraformaldehyde for 15 min on ice without (for surface protein) or with (for total protein) permeabilization with 0.1 % Triton X-100 for 5 min. Then the cells were incubated for 60 min (37 °C) with anti-Orai1 primary antibody (1:200, Abcam), washed once in PBS, and stained in 1:250 diluted CF™ 488A-labeled anti–rabbit secondary antibody (Sigma, USA) for 30 min (37 °C). Samples were immediately analyzed on a FACS Calibur flow cytometer (BD Biosciences).

### Ca^2+^ measurements

Fura-2 fluorescence was utilized to determine intracellular Ca^2+^ activity [63]. Cells were loaded with Fura-2/AM (2 μM, Invitrogen, Goettingen, Germany) for 20 min at 37 °C. Cells were excited alternatively at 340 nm and 380 nm through an objective (Fluor 40×/1.30 oil) built in an inverted phase-contrast microscope (Axiovert 100, Zeiss, Oberkochen, Germany). Emitted fluorescence intensity was recorded at 505 nm. Data were acquired using specialized computer software (Metafluor, Universal Imaging, Downingtown, USA). Cytosolic Ca^2+^ activity was estimated from the 340 nm/380 nm ratio. SOCE was determined by extracellular Ca^2+^ removal and subsequent Ca^2+^ readdition in the presence of thapsigargin (1 μM, Invitrogen) [64]. For quantification of Ca^2+^ entry, the slope (delta ratio/s) and peak (delta ratio) of Ca^2+^-entry were calculated.

Experiments were performed with Ringer solution containing (in mM): 125 NaCl, 5 KCl, 1.2 MgSO_4_, 2 CaCl_2_, 2 Na_2_HPO_4_, 32 HEPES, 5 glucose, pH 7.4. To reach nominally Ca^2+^-free conditions, experiments were performed using Ca^2+^-free Ringer solution containing (in mM): 125 NaCl, 5 KCl, 1.2 MgSO_4_, 2 Na_2_HPO_4_, 32 HEPES, 0.5 EGTA, 5 glucose, pH 7.4.

### Statistical analysis

Data are provided as means ± SEM, *n* represents the number of independent experiments. Data were tested for significance using unpaired student’s *t*-test or ANOVA as appropriate. Differences were considered statistically significant when *p*-values were < 0.05. Statistical analysis was performed with GraphPad InStat version 3.00 for Windows 95, GraphPad Software, San Diego California USA, www.graphpad.com.
